# Cold Regulation of Genes Encoding Ion Transport Systems in the Oligotrophic Bacterium Caulobacter crescentus

**DOI:** 10.1128/spectrum.00710-21

**Published:** 2021-08-25

**Authors:** Hugo L. de Araújo, Bianca P. Martins, Alexandre M. Vicente, Alan P. R. Lorenzetti, Tie Koide, Marilis V. Marques

**Affiliations:** a Departamento de Microbiologia, Instituto de Ciências Biomédicas, Universidade de São Paulo, São Paulo, Brazil; b Departamento de Bioquímica e Imunologia, Faculdade de Medicina de Ribeirão Preto, Universidade de São Paulo, Ribeirão Preto, Brazil; University of Minnesota

**Keywords:** cold adaptation, *Caulobacter crescentus*, transcriptomic analysis, cation homeostasis, potassium transport

## Abstract

In this study, we characterize the response of the free-living oligotrophic alphaproteobacterium Caulobacter crescentus to low temperatures by global transcriptomic analysis. Our results showed that 656 genes were upregulated and 619 were downregulated at least 2-fold after a temperature downshift. The identified differentially expressed genes (DEG) belong to several functional categories, notably inorganic ion transport and metabolism, and a subset of these genes had their expression confirmed by reverse transcription quantitative real-time PCR (RT-qPCR). Several genes belonging to the ferric uptake regulator (Fur) regulon were downregulated, indicating that iron homeostasis is relevant for adaptation to cold. Several upregulated genes encode proteins that interact with nucleic acids, particularly RNA: *cspA*, *cspB*, and the DEAD box RNA helicases *rhlE*, *dbpA*, and *rhlB*. Moreover, 31 small regulatory RNAs (sRNAs), including the cell cycle-regulated noncoding RNA (ncRNA) CcnA, were upregulated, indicating that posttranscriptional regulation is important for the cold stress response. Interestingly, several genes related to transport were upregulated under cold stress, including three AcrB-like cation/multidrug efflux pumps, the nitrate/nitrite transport system, and the potassium transport genes *kdpFABC*. Further characterization showed that *kdpA* is upregulated in a potassium-limited medium and at a low temperature in a SigT-independent way. *kdpA* mRNA is less stable in *rho* and *rhlE* mutant strains, but while the expression is positively regulated by RhlE, it is negatively regulated by Rho. A *kdpA*-deleted strain was generated, and its viability in response to osmotic, acidic, or cold stresses was determined. The implications of such variation in the gene expression for cold adaptation are discussed.

**IMPORTANCE** Low-temperature stress is an important factor for nucleic acid stability and must be circumvented in order to maintain the basic cell processes, such as transcription and translation. The oligotrophic lifestyle presents further challenges to ensure the proper nutrient uptake and osmotic balance in an environment of slow nutrient flow. Here, we show that in Caulobacter crescentus, the expression of the genes involved in cation transport and homeostasis is altered in response to cold, which could lead to a decrease in iron uptake and an increase in nitrogen and high-affinity potassium transport by the Kdp system. This previously uncharacterized regulation of the Kdp transporter has revealed a new mechanism for adaptation to low temperatures that may be relevant for oligotrophic bacteria.

## INTRODUCTION

Bacteria must quickly adapt to constant changes in the environment, such as nutrient starvation, oxygen availability, and abiotic stresses such as a temperature shift, which is particularly important for free-living organisms. A sudden drop in temperature causes global changes in the gene expression of mesophilic bacteria, resulting in physiological alterations that lead to a lower growth rate, including changes in the membrane lipid composition, a decrease in protein synthesis, and a change in the metabolic pathways (reviewed in references 1 and 2), which are also observed as a strategy of the psychrophiles (3). As temperature decreases, the main consequences are a decrease in membrane fluidity and increased stabilization of the secondary structures in nucleic acids, affecting important processes such as transcription and translation. Triggering the expression of a specific set of proteins, in turn, helps the cell to adjust to these unfavorable changes, mainly by increasing the fluidity of the membrane and destabilizing the secondary structures in nucleic acids (1, 2, 4).

The bacterial response to low temperatures was comprehensively studied initially in the model bacteria Escherichia coli (5) and Bacillus subtilis (6, 7). Ever since, several other species have been investigated in that context. Many of the cold-induced proteins have been identified and shown to be essential for cellular adaptation to low temperatures (8). The importance of keeping the functionality of RNA molecules can be demonstrated by the ubiquitous cold induction of RNA-modifying enzymes in all domains of life. Small cold shock proteins (CSPs), DEAD box RNA helicases, and RNases have been shown to play a critical role in the cold shock response of Gram-negative (1, 9–11) and Gram-positive (12–15) bacteria, a conserved feature in Archaea (16) and plants (17). CSPs possess a conserved cold shock domain, composed of two nucleic acid-binding motifs in tandem that bind to RNA and prevent the formation of secondary structures (18–21). These proteins have been shown to participate in many cellular processes, such as transcription antitermination and the initiation of translation (22, 23). The DEAD box RNA helicases unwind the RNA:RNA bonds in the RNA secondary structures and are frequently found associated with the RNA degradosome complex, where they play a role in RNA decay and are also important for the correct assembly of the rRNA-protein complexes (24–28).

The response to temperature downshift frequently overlaps with the response to other stresses, indicating a more universal stress response. In cyanobacteria, it has been reported that cold-induced genes also respond to other stresses, such as high osmolarity, oxidative and acidic stresses, or redox perturbations (29). The pre-exposure to low temperature can increase the tolerance to other stresses, which indicates that the alteration in gene expression induced by cold may prepare the cells to withstand different severe situations (30, 31). Moreover, an interesting overlap between cold and hyperosmotic shock responses has been reported, showing that the accumulation of compatible solutes can increase cryoprotection (32, 33).

The cold shock genes are mainly regulated posttranscriptionally; mRNA stabilization and translation efficiency are differentially affected according to temperature. These mechanisms have been mostly studied for the CSP genes, in which the long 5′ untranslated region (UTR) was shown to have a role in *cspA* and *cspB* mRNA stability and translation (34–36). The CSP mRNAs contain *cis* elements that favor translation initiation at low temperatures, while other mRNAs are transiently inefficiently translated under these conditions, until the ribosomes become cold adapted by association with other factors (1). The CSPs, in turn, have a role in facilitating the transcription and translation of other highly structured mRNAs.

The alphaproteobacterium genus *Caulobacter* comprises free-living mesophiles and oligotrophs, widespread in humid soils associated with plants and in aquatic environments that can freeze for long periods of time (37, 38). The remarkable ability of Caulobacter crescentus NA1000 to withstand freezing is stimulated by nonfreezing temperatures, indicating that its cryotolerance can be induced by low temperatures (39). The genome of C. crescentus NA1000 encodes four paralogs of CSPs, two cold-induced (*cspA* and *cspB*) and the other two induced at stationary phase (*cspC* and *cspD*) (40). Three DEAD box RNA helicases are present: RhlE, DbpA, and RhlB, the latter being part of the RNA degradosome complex (41). The involvement of several of these proteins with cold stress resistance has been reported by previous studies (36, 39, 42). The *cspA* and *cspA*/*cspB* mutants present a more severe growth phenotype at 10°C than that of the *cspB* null mutant, suggesting that CspA may be more important to cold adaptation than CspB (36). The C. crescentus *rhlE* null mutant is deficient in growth at low temperatures and survival to freezing (39), and *rhlE* is upregulated at low temperatures due to both transcriptional and posttranscriptional mechanisms (42). Moreover, a change in the RNA degradosome composition was observed at low temperatures, when other proteins become attached to the main complex, including RhlE and Rho (42). These studies have identified a few genes important for cold adaptation, but they were mostly related to the physiological response toward the increase of RNA secondary structure stability. The study hereby presented aimed to identify other cold-regulated genes that could unveil a comprehensive picture of physiological adaptations to low temperatures in the oligotroph C. crescentus.

Although much is known about the cold stress response in many genera, scarce information is available regarding the strategies utilized by the alphaproteobacteria group. Therefore, a comprehensive analysis of the C. crescentus cold-induced genes could provide a picture of how this oligotrophic alphaproteobacterium responds to low-temperature stress. In this study, we used a global transcriptomic analysis (RNA-Seq) approach to characterize the C. crescentus response to low temperatures. This strategy allowed us to identify the differentially expressed genes (DEG) encoding proteins involved in a wide range of biological processes. In particular, a predominant category is the transmembrane transport of cations, with an expressive downregulation of the iron transporters and upregulation of the assimilatory nitrate reduction pathway and of the Kdp potassium uptake system, indicating that Fe^2+^, NH_4_^+^, and K^+^ homeostasis is relevant for this response. The basis for the cold induction of the *kdp* operon was investigated, showing that the DEAD box RNA helicase RhlE and the transcription termination factor Rho have opposite roles in maintaining the levels of *kdp* mRNA. Moreover, several small regulatory RNAs (sRNAs) were upregulated, indicating that posttranscriptional control is relevant for the regulation of cold stress response.

## RESULTS

### Global expression analysis.

**FIG 1:**
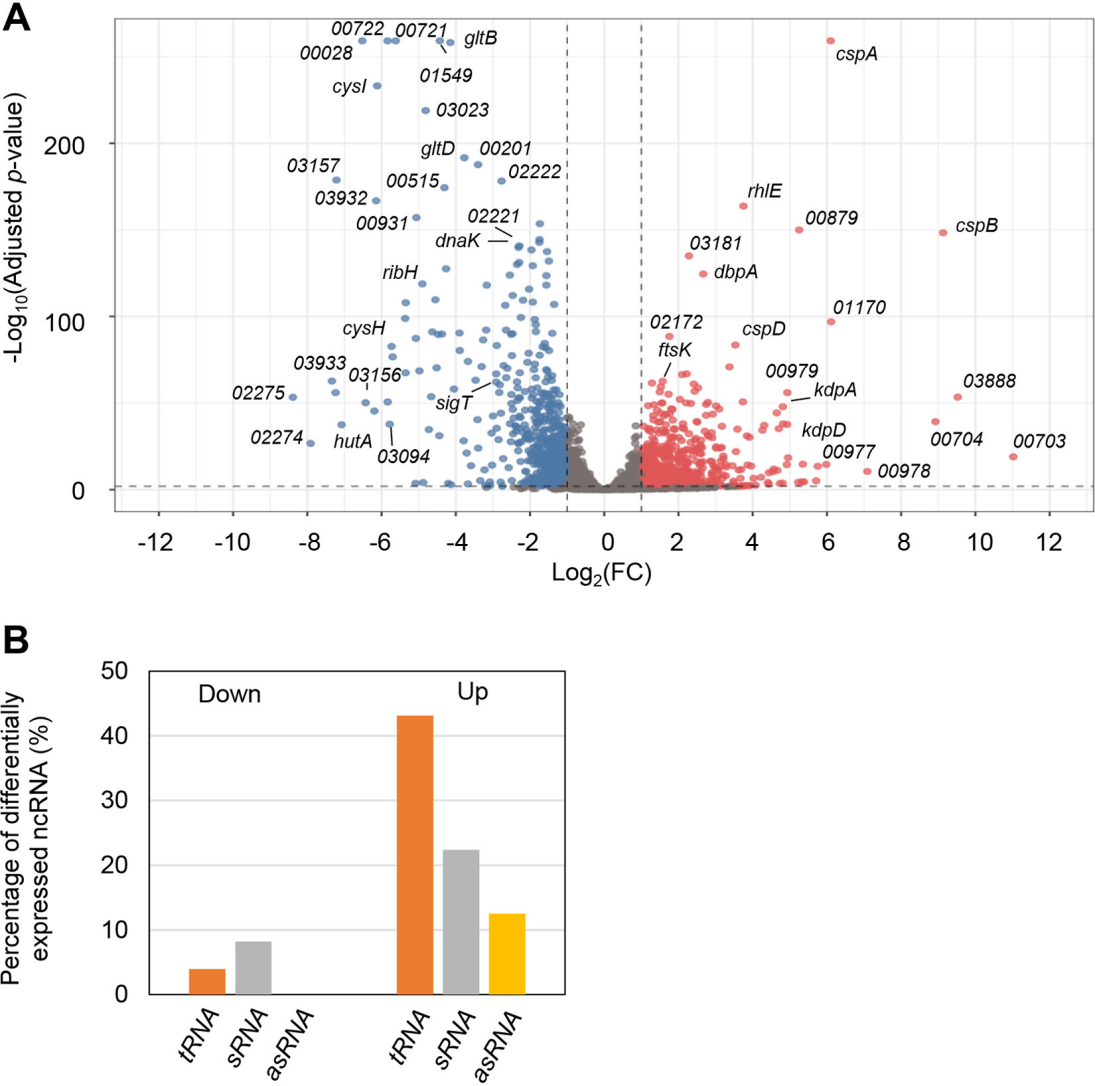
Genes that are differentially expressed during cold stress. (A) Volcano plot for the transcriptomic analysis of C. crescentus under cold stress. The genes represented by red dots are those that met the cutoff criteria of a log_2_ fold change of ≥1 and those by blue dots of ≤-1 and an adjusted *P* value of <0.01, while those represented by the gray dots did not meet the cutoff criteria. Dots corresponding to several of the most DEG, or the genes of interest, were labeled with the respective gene name as indicated. Genes satisfying the condition of adjusted *P* value = 0 had these values replaced by the smallest nonzero adjusted *P* value multiplied by 10^−2^. (B) Percentage of differentially expressed noncoding RNAs (ncRNA) that were decreased (Down) or increased (Up) in abundance (10°C/30°C). The percentage is relative to the total number of ncRNA in each category.

The response to temperature decrease in bacteria was previously characterized and develops in a two-step manner: an immediate response (called the acclimation phase), when there is a transient arrest of cell growth, followed by an adaptation phase, when cells resume growth (9). It was previously determined that in C. crescentus NA1000, the growth lag period following cold shock is about 4 h (39), but after 2 h the synthesis of most cold shock proteins is back to their initial levels (36). Therefore, to identify the genes differentially expressed in response to cold stress, we compared the transcriptome of the wild-type C. crescentus strain NA1000 under optimal growth conditions (30°C) and after 2 h under cold stress (10°C). Our results showed that 656 genes were upregulated and 619 were downregulated with a log_2_ fold change of ≥1 and ≤–1, respectively (Fig. 1A, Table S1 in the supplemental material). Of these, 66 genes for noncoding RNAs (ncRNAs) were differentially expressed, encoding tRNAs, small noncoding RNAs (sRNAs), and antisense RNA (asRNAs) (Fig. 1B). Interestingly, a higher percentage of the tRNAs and sRNAs was found among the upregulated genes.

**FIG 2:**
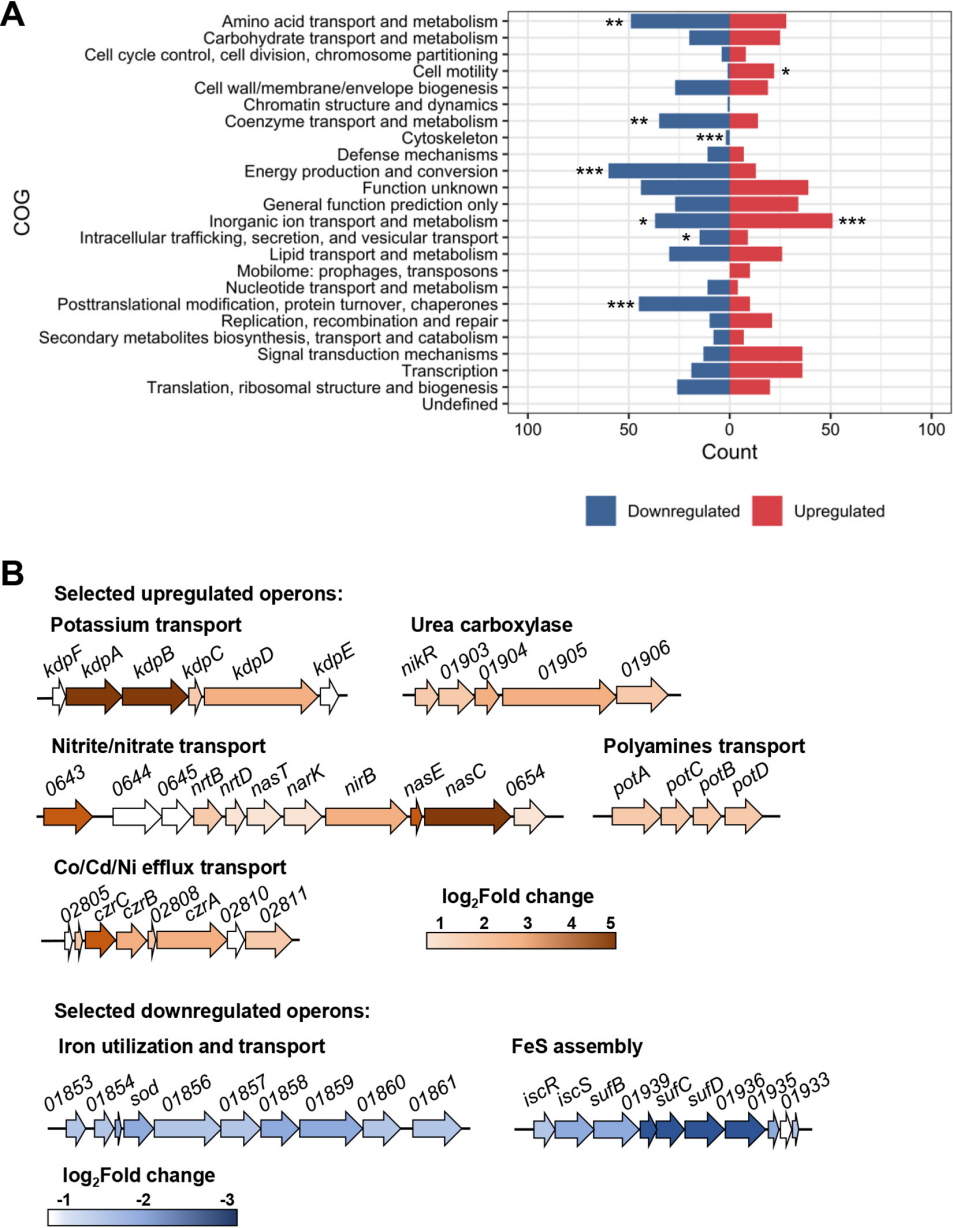
Functional characterization of the DEG. (A) Enrichment of functional categories among DEG. The total number of genes belonging to distinct orthologous groups of encoded proteins (COG) was analyzed to verify whether the representation of each COG within the up- or downregulated groups was significant against the total gene background. The categories defined as being prominent in each group are those with an adjusted *P* value of <0.05, as indicated: *adjusted *P* value < 0.05; **adjusted *P* value < 0.01; ***adjusted *P* value < 0.001. (B) Graphic representation of selected up- and downregulated operons. Most of the operons encode transport systems. The levels of expression are color-coded approximately to the log_2_ fold change of each gene.

The Clusters of Orthologous Groups of proteins (COG) were used to identify the functional categories of the DEG (Fig. 2A, Table S1). An analysis of the enrichment of categories within the up- and downregulated groups was carried out, and the results showed that the inorganic ion transport and metabolism category was significantly enriched in both groups (Fig. 2A). Furthermore, a few other categories were enriched among the downregulated genes: amino acid transport and metabolism; coenzyme transport and metabolism; energy production and conversion; intracellular trafficking, secretion, and vesicular transport; and posttranslational modification, protein turnover, and chaperones. Regarding the set of upregulated genes, the cell motility category was the only one to be overrepresented besides inorganic ion transport and metabolism.

**FIG 3:**
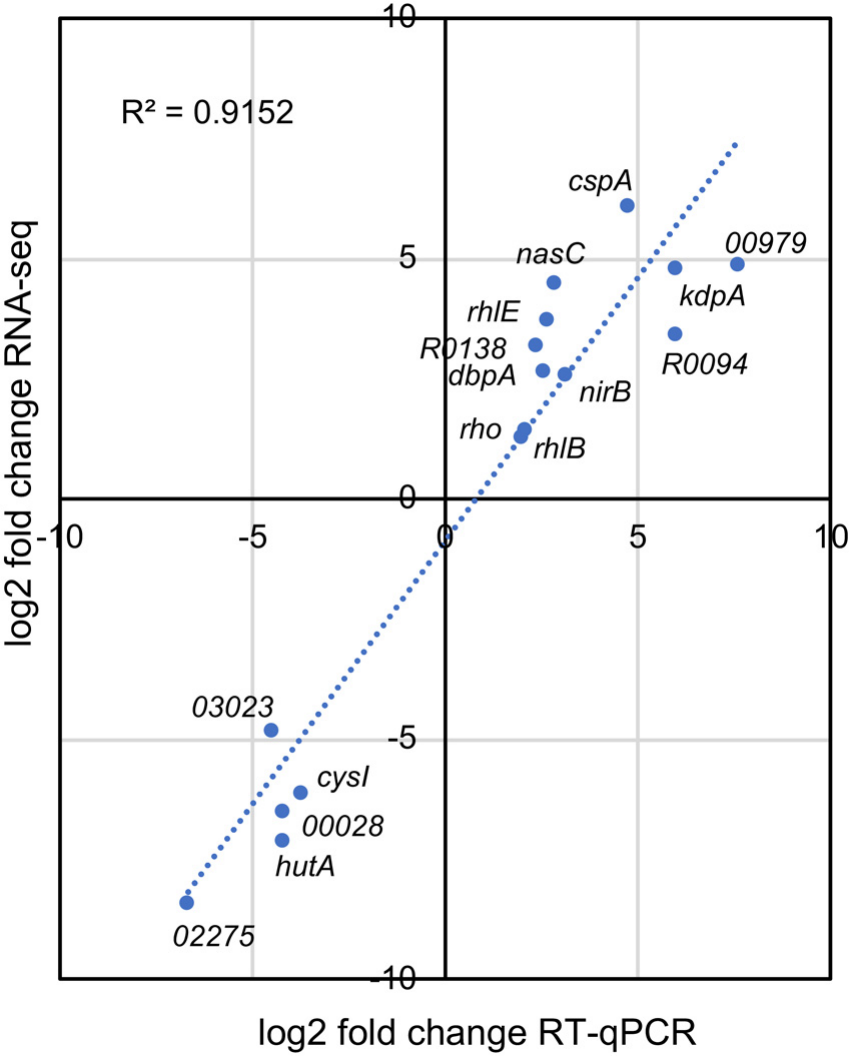
Validation of RNA-Seq data by RT-qPCR analysis. The relative expression was determined by RT-qPCR from the total RNA of cells incubated at 10°C for 2 h, relative to each gene expression at 30°C. The log_2_ fold change in the RT-qPCR data was plotted against the log_2_ fold change obtained from the RNA-Seq data. The expression levels for selected genes were plotted, and the identification of each gene is indicated.

A more detailed analysis of the genes belonging to the transport category showed that the DEG were organized into large operons that were coregulated (Fig. 2B). We measured the expression of 11 upregulated and 5 downregulated genes by reverse transcription quantitative real-time PCR (RT-qPCR), and the results were consistent with the pattern of expression observed in the RNA-Seq experiments (Fig. 3), validating the transcriptome analysis. Some of the most relevant DEG will be discussed below.

### Iron and sulfur uptake and central metabolism genes are downregulated under cold stress.

As discussed above, the genes encoding enzymes for the central energy metabolism (belonging to the energy production and conversion category) were downregulated at low temperatures, leading the cells to enter a slow-growth phase compatible with the new condition. All the genes encoding enzymes of the glycolysis/gluconeogenesis, citrate cycle, pentose phosphate, and oxidative phosphorylation pathways were downregulated (Table S1). Furthermore, the genes encoding assimilatory sulfate reduction and cysteine and methionine synthesis were strongly downregulated.

**FIG 4:**
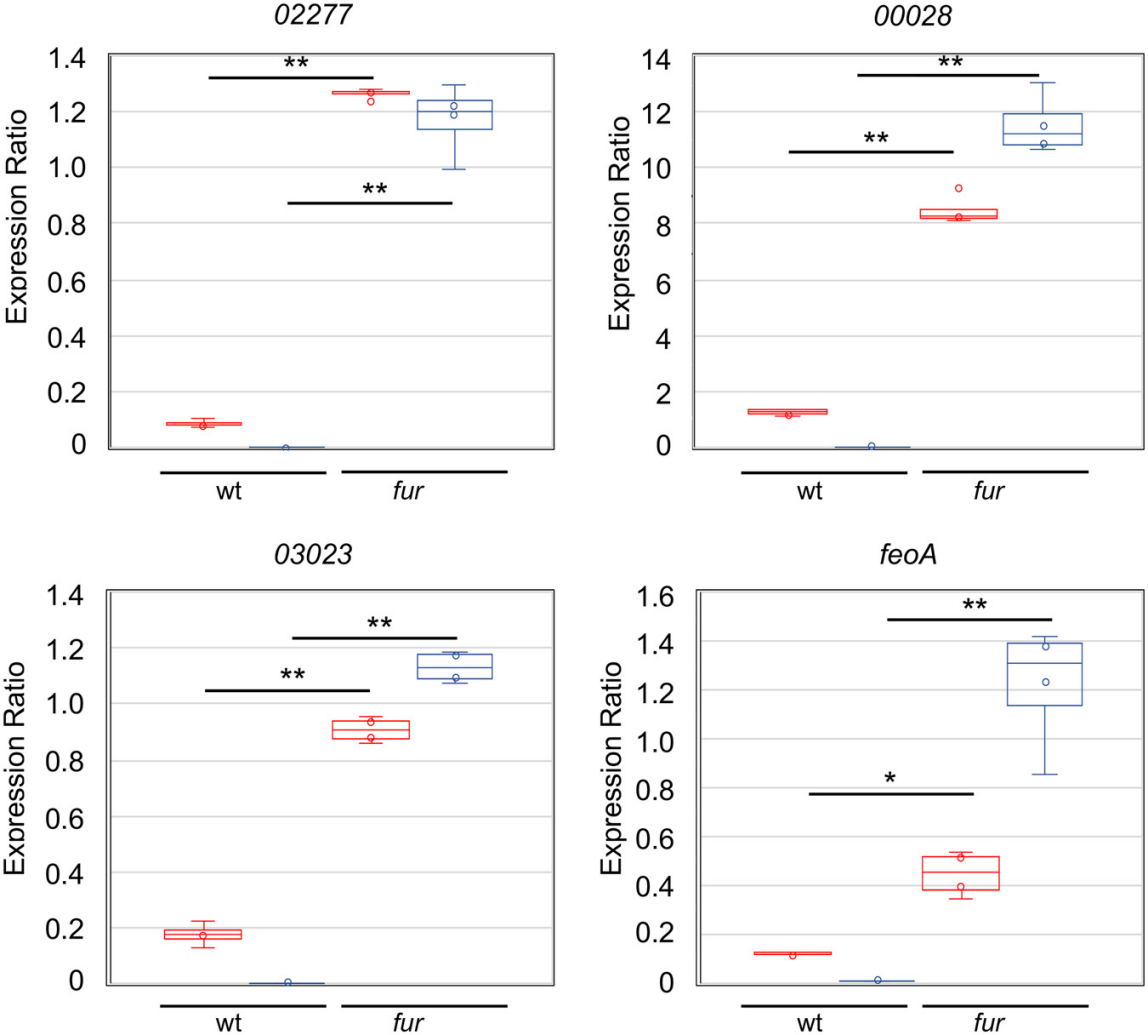
mRNA levels of Fur-regulated genes in C. crescentus. The expression was determined by RT-qPCR from the total RNA isolated from wild-type strain NA1000 (wt) or from the *fur* strain cultures growing in M2 medium, at 30°C (red) and after 2h at 10°C (blue). The dots indicate the individual results; the central bar indicates the mean, and the vertical bars indicate the standard error. The significance was calculated using Student’s *t* test (*P < 0.05, **P < 0.001).

Interestingly, many of the most downregulated genes are involved in iron metabolism and are regulated by the ferric uptake regulator (Fur) (43). This was observed mostly for those negatively regulated, such as the iron transporters *feoAB*, *hutA*, CCNA_00028, CCNA_00138, and CCNA_03023; the bacterioferritin-associated ferredoxin CCNA_03372; the riboflavin synthesis operon CCNA_00929-0932; and the Fe-S cluster biogenesis operon CCNA_03156-3159 (Fig. 2B) (43, 44). In order to verify whether downregulation of these genes was Fur-dependent, RT-qPCR was carried out for four genes directly repressed by Fur (43, 44) in the wt and *fur* mutant, at 30°C and 10°C (Fig. 4). The results showed that while the relative expression was much higher in the *fur* mutant, as expected, downregulation was no longer observed at 10°C, and in fact expression increased (CCNA_00028, CCNA_03023, and *feoA*) or did not change (CCNA_02277). These results suggest that Fur is mediating the cold-induced downregulation of its regulon, perhaps due to changes in its affinity for iron or for its operator. Other Fur-independent genes were also severely downregulated, such as the *groESL* operon, which is upregulated in response to iron limitation (43), indicating that this effect could be related mostly to iron levels. The downregulation of the protein chaperones GroELS and DnaK also indicates that protein homeostasis is maintained at cold temperatures and that the sigma32-dependent heat shock response is turned off, agreeing with the lower protein synthesis activity.

The genes encoding the general stress extracellular function (ECF) sigma factors *sigT* and *sigU* were also downregulated, along with their coregulators NepR, PhyK, PhyR, and the regulatory RNA GsrN, indicating that the adaptation to low temperatures does not trigger a general stress response (45).

### Upregulation of genes encoding RNA-binding proteins aids in coping with RNA secondary structures.

The most upregulated genes in response to cold stress encode proteins that interact with nucleic acids, notably RNA (Table S1). In particular, a pronounced increase in the expression of *cspA*, *cspB*, and *rhlE* was observed. C. crescentus NA1000 has four cold shock proteins (CSPs), CspA, CspB, CspC, and CspD. The cold-induced CspA and CspB have been reported to be essential for cold stress survival, while the stationary-phase-induced CspC and CspD are involved in adaptation to long starvation periods (36, 40, 46). While *cspA*, *cspB*, and *cspD* were among the cold-induced genes in our analysis, the levels of induction were distinct, with *cspB* being the most highly differentially expressed of all genes (560-fold), *cspA* presenting a 68-fold increase and *cspD* only an 11-fold increase. This high level of *cspB* expression was also observed for the genes downstream (CCNA_03888, CCNA_00703, and CCNA_00704) (see Fig. S1 in the supplemental material). These genes are transcribed independently from *cspB* and also encode small proteins (65 to 119 amino acids) with no sequence similarity. Interestingly, CCNA_03888 encodes a conserved basic protein (predicted pI = 10.6) and is syntenic with *cspB* in several genera of the alphaproteobacteria group (data not shown). The role of these small proteins in the cold-stress response is still unknown.

The genes for the DEAD box RNA helicases RhlE, DbpA, and RhlB and the transcription terminator factor Rho were also induced. The RNA helicase RhlE was already described as important for cold adaptation in C. crescentus, since the mutant *rhlE* is extremely sensitive to low temperatures (39, 42). RhlB is the main helicase associated with the RNA degradosome, but RhlE and DbpA were shown to also be associated with it at low temperatures (42). The DEAD box RNA helicases’ role in unwinding short RNA secondary structures may provide more effective degradation of highly structured mRNAs and therefore have an important role in mRNA stabilization under cold stress.

### Upregulated noncoding RNAs indicate a posttranscriptional layer of regulation.

While 11 noncoding small regulatory RNAs (sRNAs) were downregulated, 31 sRNAs were upregulated at low temperatures at least 2-fold (Fig. 1B, Table S1). sRNAs act as posttranscriptional regulators, and several studies on their roles in environmental stress responses have been performed over the last decades (47–49). Although the gene for the RNA chaperone Hfq was not differentially expressed, previous studies have shown that it participates in this type of regulation, acting as an intermediate between the sRNA and its target (50, 51). Among the most upregulated sRNAs is the cell cycle-regulated CcnA (R0094) (11-fold), which is responsible for controlling two master regulators of the cell cycle, CtrA and GcrA (52). As the overexpression of CcnA leads to a slow growth phenotype (52), it is tempting to speculate that it contributes to the decrease in growth rates observed at low temperatures.

While 22 tRNAs were upregulated under our conditions, only two were downregulated (Fig. 1B). The complex regulation of protein synthesis during adaptation to stress is also associated with changes in the tRNA pool of the cell and the utilization of unusual codons (53, 54). However, the altered tRNAs in our analysis predominantly correspond to the expected codon usage preference of *Caulobacter*.

### Upregulated genes for transport systems for detoxification and cation homeostasis.

Several genes related to transport and membrane components were upregulated at low temperatures, belonging to the secretion systems, lipid metabolism, TonB-dependent receptors, and flagellum assembly categories. Under cold stress, the membrane suffers from rigidification of its structure, and the cell needs to adapt its components to maintain its membrane fluidity and functions (2). Interestingly, however, very few DEG related to lipid metabolism and transport seem to be specifically associated with processes involved in countermeasures to the effects of cold on the membrane. In fact, only two genes encoding a fatty acid desaturase (CCNA_03535) and a sterol desaturase (CCNA_01743) were upregulated. Among those encoding transport systems, three genes encoding proteins belonging to the AcrB-like cation/multidrug efflux pump (CCNA_00850, CCNA_02809, and CCNA_03219) were upregulated. These proteins are part of the resistance/nodulation/division (RND) family and are involved in the export of cations or drugs. In fact, the upregulated *czrCBA* (CCNA_02806-09) operon (Fig. 2B) encodes an RND efflux system for cadmium/zinc/cobalt (55), suggesting that the detoxification of cations is an important response to low temperature conditions.

Nitrogen is one of the most important nutrients and also performs a regulatory role in modulating cell cycle progression via the regulation of (p)ppGpp accumulation (56), and C. crescentus cells use nitrate (NO_3_-) and ammonium (NH_4_^+^) as nitrogen sources (57). The upregulation of the genes encoding nitrogen regulatory proteins, the sigma factor RpoN, histidine kinase NtrB, and the nitrogen assimilation response regulator NtrC, agrees with the upregulation of many genes belonging to the ammonium limitation stimulon (57). The operon encoding the nitrate/nitrite transport systems *nrtABD* and *narK-nirB-nasE-nasC* was upregulated in our analysis (Fig. 2B). The Nrt ABC transporter and the NarK protein transport NO_3_- and NO_2_-, respectively, from the extracellular medium, and subsequently NasC converts NO_3_- into NO_2_-. Nitrite is then converted into NH_4_^+^ by NirB in the assimilatory nitrate reduction pathway. However, only one glutamine synthetase gene, *glnA2*, was upregulated, and the glutamate synthase genes (*gltB* and *gltD*) were highly downregulated, indicating that not all NH_4_^+^ may be incorporated into amino acids. Since the M2 medium provides sufficient ammonium chloride and the cultures are not under nitrogen deprivation, these data suggest that the upregulation of nitrogen metabolism could be providing an increase in the NH_4_^+^ concentration in the cytosol. In fact, other expression profiles of genes that could be involved in this response agree with this idea: the operon encoding the polyamine uptake system (putrescine/spermidine transport CCNA_03235-03239) was also upregulated, indicating the need to increase the uptake of NH_4_^+^; and the operon encoding urea-carboxylase (CCNA_01902-06), which regulates intracellular nickel concentration and was implicated in acid response in *Helicobacter* (58), was also upregulated (Fig. 2B). The expression of this operon could also be regulated by nickel ions, since urease is a nickel-binding protein, agreeing with the upregulation of the RND cation export system.

### The Kdp potassium transporter is highly upregulated during cold stress.

The *kdp* operon, encoding a high-affinity potassium transporter system, was also upregulated at low temperatures, except for *kdpE*, which was not differentially expressed. However, a closer inspection identified a nonannotated gene upstream of *kdpA* that was also upregulated. This gene codes for a protein with high similarity to E. coli KdpF (Fig. S2A, B), so it is likely that in C. crescentus the operon *kdpFABCDE* is organized similarly as in other bacteria.

Upstream of *kdpF*, there is a σ^T^-dependent promoter, and SigT is important for the induction of the operon under osmotic stress (45). Although the transcription start site (TSS) upstream of *kdpF* has not been determined, a putative TSS upstream of *kdpC* was identified, and the levels of expression of *kdpCDE* were higher than those for *kdpFAB* (59), suggesting that they are also independently regulated. Transcription from the σ^T^-dependent promoter to the start codon of KdpF would generate a 5′ UTR comprising about 100 nucleotides that is predicted to be highly structured (Fig. S2C). This could reflect in posttranscriptional regulation of the *kdp* operon, altering the mRNA half-life or its translation, depending on the secondary structure.

**FIG 5:**
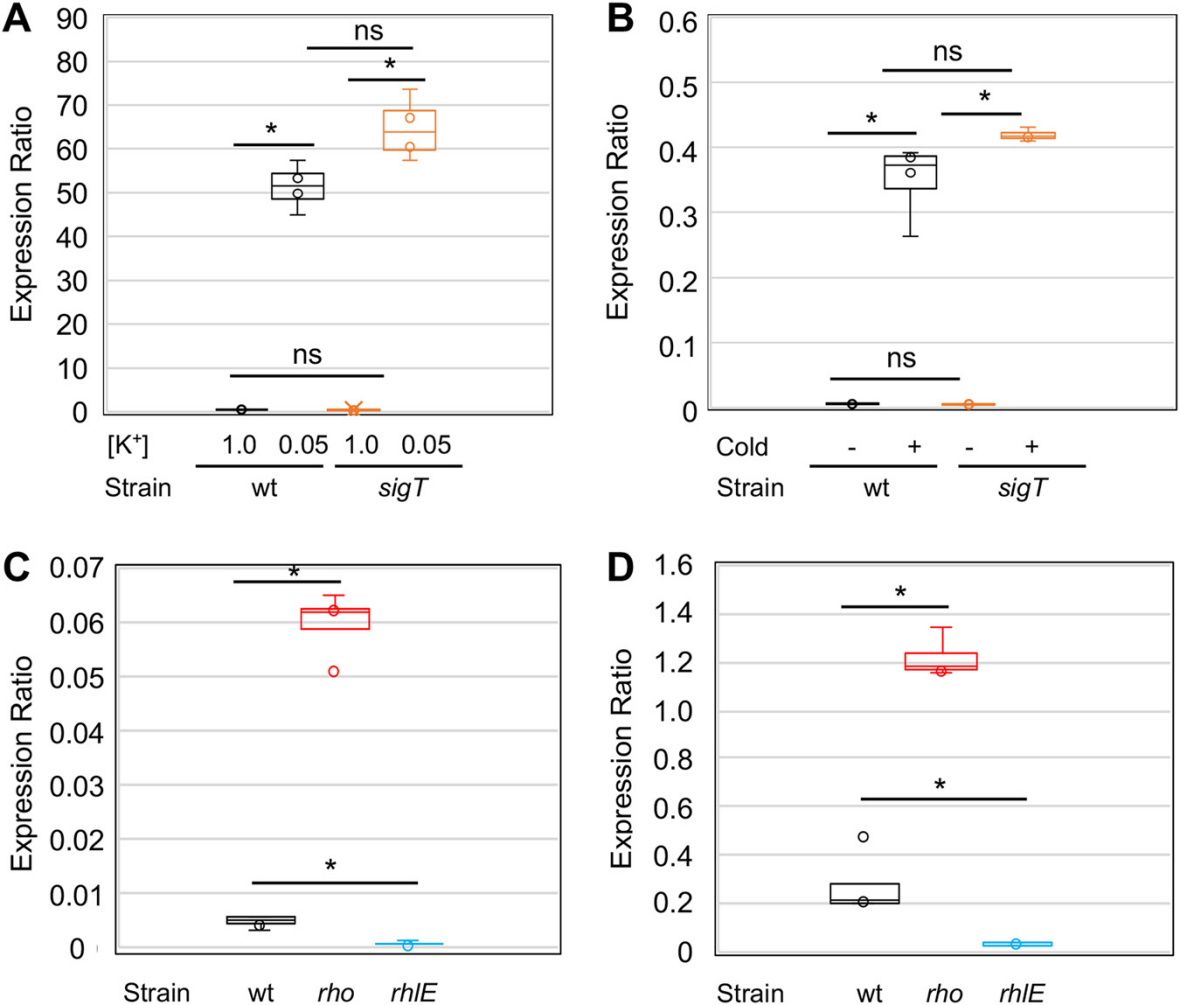
*kdpA* mRNA levels in C. crescentus. The expression was determined by RT-qPCR from the total RNA isolated from cultures growing in M2 medium, calculated as 2^-ΔΔCT^. (A) Expression was measured either in the wild-type (wt) strain NA1000 or in the *sigT* strain using total RNA from cultures incubated for 2 h in sodium phosphate-based M2 medium (M2-K) with sufficient (1 mM KCl) or deficient (50 μM KCl) potassium levels. The expression was determined relative to the respective culture in the K^+^-sufficient condition. (B) Expression was measured either in the wild-type strain NA1000 (wt) or in the *sigT* strain using the total RNA from cultures incubated in M2 medium at 30°C (Cold -) and after 2 h at 10°C (Cold +). The expression was determined relative to the wt strain at 30°C. (C) Expression from cells grown in M2 medium at 30°C from the wild-type strain (wt) and in the *rho* mutant strain (NA1000 *rho*::Tn5) and *rhlE* mutant strain (NA1000 *rhlE*::Tn5) (Table S2). (D) The same as in part C, but cultures were grown at 10°C. The results shown are from 4 independent experiments using RNA from 2 biological replicas. The significance was calculated using Student’s *t* test (*P < 0.001).

The upregulation of the *kdp* operon at low temperatures was intriguing, so we further analyzed its regulation in order to understand the molecular basis for the cold induction. Since this operon should respond to low K^+^ levels, we determined its expression in response to low [K^+^] concentrations and low temperatures, both in the wild-type strain NA1000 and in the *sigT* mutant strain. For the response to low [K^+^], *kdpA* expression was determined by RT-qPCR, using RNA isolated from cells grown under different [K^+^] in a Na^+^ phosphate-buffered M2 medium with increasing concentrations of KCl added. The results showed that the gene is highly induced by low [K^+^] both in the wt strain and in the *sigT* mutant (Fig. 5A), indicating that upregulation in this case does not require transcription from the SigT promoter. The same experiment was carried out using RNA from exponentially growing cells in M2 medium that were either incubated at 30°C or after 2 h at 10°C, and *kdpA* cold induction was also SigT-independent (Fig. 5B). In order to confirm that the observed cold induction was not restricted to the minimal medium condition, we determined the *kdpA* expression in rich peptone-yeast extract (PYE) medium at both temperatures (Fig. S3), and the results showed that it was induced to the same extent as in minimal medium. Therefore, the cold induction of *kdpA* might be regulated by a putative second promoter and/or by posttranscriptional mechanisms such as transcript stabilization.

The RNA helicase RhlE and the transcription terminator Rho are induced during cold stress and probably have a role in the cold-adapted RNA degradosome (42). To investigate the role of Rho and RhlE in *kdp* expression, the expression of *kdpA* was determined in the C. crescentus *rhlE* and *rho* mutant strains by RT-qPCR (Fig. 5C and D). We found that, although the levels of *kdpA* mRNA were still higher at low temperatures in both strains (about 20-fold and 30-fold in the *rho* and *rhlE* mutant strains, respectively), the steady-state transcript levels were very different from those in the wt strain. The *kdpA* transcript levels were 5- to 10-fold higher in the *rho* mutant and lower to the same extent in the *rhlE* mutant at either temperature.

**FIG 6:**
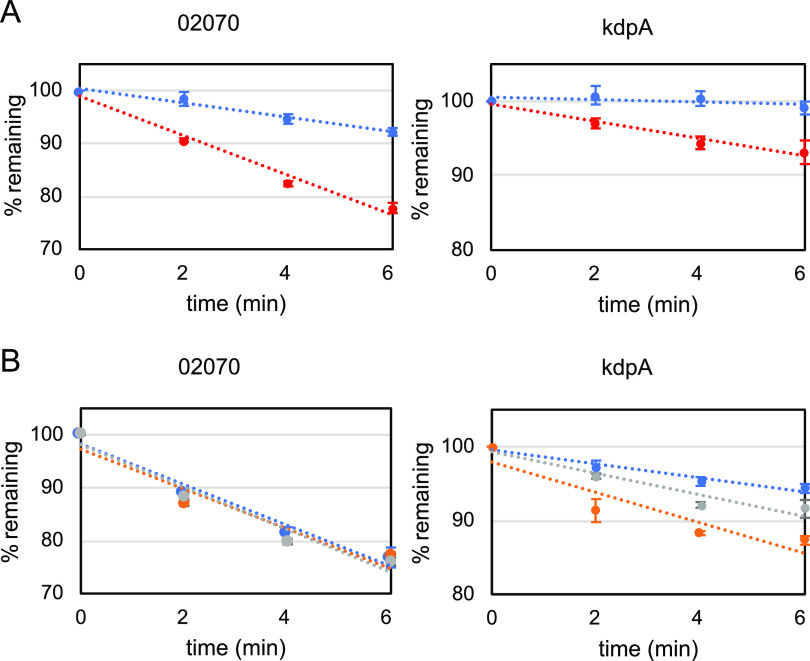
mRNA decay of *kdpA*. (A) C. crescentus NA1000 (wt) was grown in M2 medium at 30°C (red) or after 2 h at 10°C (blue), followed by treatment with 200 μg/ml rifampin. At the indicated times before (*t* = 0) and after the addition of rifampin, the cells were collected and the total RNA was extracted from 2 biological replicas. (B) C. crescentus NA1000 (blue), *rho* (orange), and *rhlE* (gray) strains were grown in M2 medium at 30°C, followed by treatment with 200 μg/ml rifampin. At the indicated times before (*t* = 0) and after the addition of rifampin, the cells were collected and the total RNA was extracted from 2 biological replicas. The mRNA levels of *CCNA_02070* (control) and *kdpA* were determined relative to *t* = 0 by RT-qPCR using primer pairs for the amplification of each gene. The results shown are from 4 independent experiments using RNA from 2 biological replicas. The dotted lines indicate the tendency lines. The vertical bars indicate the standard deviation.

To investigate whether different mRNA stability could account for these differences, an mRNA decay assay was carried out in the wt strain grown at 30°C and 10°C (Fig. 6A). The results showed that both the control CCNA_02070 mRNA, which is not cold-induced, and the *kdpA* mRNA were stabilized at 10°C. However, the steady-state mRNA levels (defined by the expression ratio) of *kdpA* were much higher at 10°C (Fig. 5B), while those of CCNA_02070 were the same under both conditions. Moreover, mRNA decay was assessed in the wt, *rho*, and *rhlE* strains at 30°C (Fig. 6B), and the results showed that while the control mRNA decayed at a constant rate in all strains, the *kdpA* mRNA had a more pronounced decay rate in the *rho* and *rhlE* strains. These results indicate that mRNA stabilization could partly account for the increase in the expression of *kdpA* at 10°C. RhlE is required for the stabilization of the *kdp* mRNA and therefore for maximal levels of expression, perhaps by interacting with the highly structured 5′ region of the *kdp* operon.

Surprisingly, the *kdpA* mRNA was also less stable in the *rho* mutant, despite the fact that Rho negatively regulates *kdpA* expression. One explanation for this effect on *kdpA* expression would be Rho-mediated transcription termination, since the *kdpA* transcript levels are much higher in the *rho* mutant. Interestingly, analysis of the RNA-Seq data for the *kdp* operon showed an abrupt decrease of the reads immediately after the *kdpF* gene (more visible at 10°C) (Fig. S4). This could be explained either by premature transcription termination or by cleavage followed by the destabilization of the downstream transcript. Although this region of the mRNA is highly structured, we did not find any putative intrinsic terminator, suggesting that if transcription attenuation is occurring, it might be mediated by Rho.

**FIG 7:**
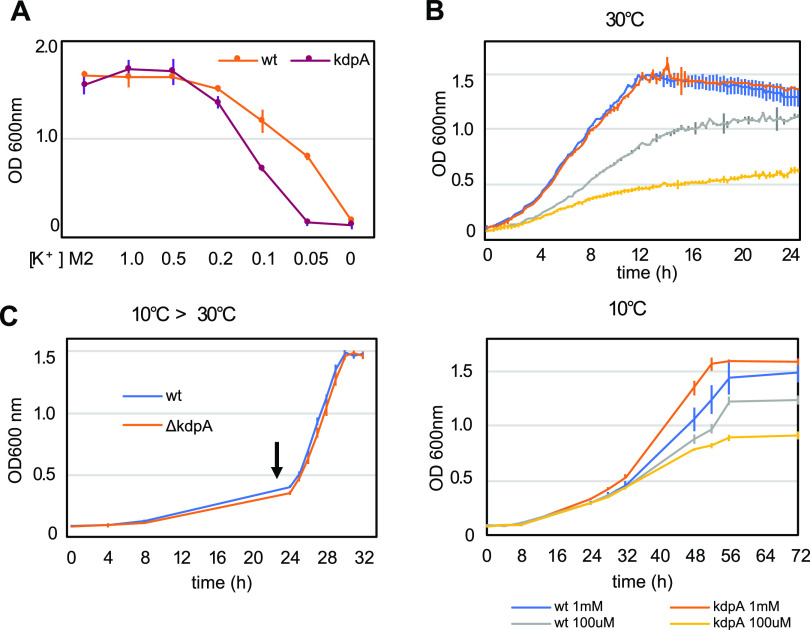
Phenotypic characterization of the *kdpA* mutant. (A) Growth of the wild-type strain and *kdpA* mutant in different potassium concentrations. Cells from ON cultures were washed with M2-K and inoculated into M2 medium or M2-K with increasing concentrations of KCl added as indicated. The growth was determined by measuring the OD_600_ of the cultures after 24 h at 30°C with agitation. [K^+^] is indicated in mM; M2, standard M2 minimal medium. (B) Growth of the wild-type strain and *kdpA* mutant in M2-K with either 100 μM or 1 mM KCl, incubated at 30°C or 10°C with agitation. (C) Cultures of the wild-type strain and *kdpA* mutant were grown in M2 medium at 10°C for 24 h and then transferred to 30°C at the time indicated (arrow). The lines indicate the averages and the vertical bars the standard deviation for each time point. The growth at 30°C was measured every 15 min and at 10°C every 4 h at the indicated time points.

In order to investigate whether the potassium transport mediated by KdpA is important for response to low temperatures and other stresses, a strain with an in-frame deletion of *kdpA* was generated (Fig. S5) and phenotypically characterized. As expected, the *kdpA* strain showed a growth phenotype only in low [K^+^] (Fig. 7A). This difference in growth can be observed both at 30°C and at 15°C (Fig. 7B), indicating that this phenotype is not aggravated at low temperature. To verify whether the mutant could have a defect in the resumption of growth after cold stress, the cultures were grown at 10°C for 24 h and then transferred to 30°C (Fig. 7C), and no difference was observed in the growth rate. Moreover, the *kdpA* mutant phenotype was similar to that of the wt strain in response to osmotic stress generated by the addition of either 85 mM NaCl or 150 mM sucrose and to acidic pH (4.0) (Fig. S6). The resistance to low temperature was assessed by incubating the plates at 15°C for 5 days or freezing at –20°C before plating, and the results showed that the *kdpA* mutant presented no phenotypic alteration compared to the wt strain (Fig. S6). These results indicate that despite the *kdp* operon being highly induced at low temperatures, K^+^ uptake mediated by KdpA is not essential for growth at low temperatures, but it may contribute to the overall osmotic balance of the cell under these conditions.

## DISCUSSION

The study of how bacteria cope with the physiological alterations caused by low temperature stress is of significance for understanding the different strategies used for each group. Although responses are common to several bacteria, such as those regarding RNA metabolism, other responses are particular to each group and are related to their way of life. C. crescentus is unique in this respect. It is a free-living alphaproteobacterium that thrives in oligotrophic aquatic environments and therefore severely suffers the effects of temperature change. This study has unveiled the strategies C. crescentus uses to maintain cell homeostasis and showed that the regulation of cation homeostasis is important for this response.

Bacteria have diverse mechanisms to overcome the cold stress effect in membranes, including incorporating desaturated fatty acids (2, 60). In E. coli, this adaptation occurs mainly by the incorporation of a monounsaturated fatty acid, via the cold-induced acyltransferase LpxP (61). B. subtilis and cyanobacteria seem to utilize similar approaches, but instead of introducing unsaturated fatty acids, they desaturate the current ones in the membrane by upregulating *desKR* and *desABD*, respectively (29, 62). It was proposed that C. crescentus can incorporate exogenous fatty acids into phospholipids and utilizes a similar synthesis pathway of unsaturated fatty acids as E. coli, via the β-hydroxydecanoyl thioester dehydrase (63). Moreover, genes encoding a fatty acid desaturase (CCNA_03535) and a sterol desaturase (CCNA_01743) were upregulated and perhaps have a role in adjusting the membrane lipid saturation in response to cold.

As expected, the cold shock genes *cspA* and *cspB* were highly induced, but surprisingly the stationary-phase-induced *cspD* gene was also upregulated. C. crescentus *cspD* is induced by carbon starvation via ppGpp but also responds to a decline in the growth rate, which is also observed after cold stress (64). The genes encoding components of the RNA degradosome, *rlhB*, and the genes for PNPase and RNase D were upregulated, as well as the DEAD box RNA helicases RhlE and DbpA and the transcription terminator Rho, as described for other bacteria (1).

**FIG 8:**
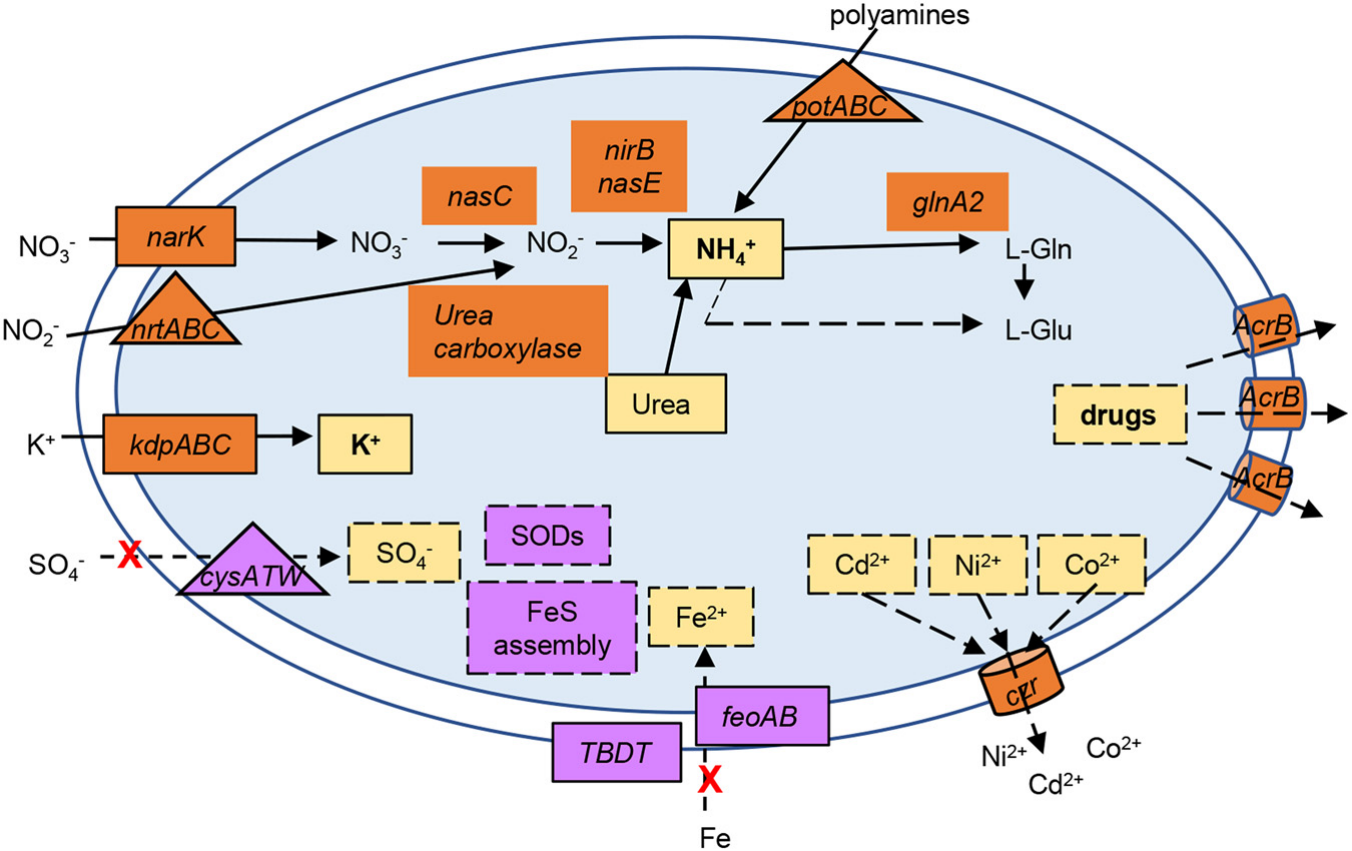
Overview of the putative alterations in ion homeostasis upon cold stress. The ion transport systems or converting enzymes whose expression was upregulated (in orange) or downregulated (in purple) at 10°C compared to 30°C are indicated. The solid lines indicate a putative increase in the intracellular concentration, while the dashed lines indicate a putative decrease in the intracellular concentration of a given ion or molecule. The red X indicates a decrease of transport; the triangles indicate ABC-type transport systems, and the cylinders indicate RND efflux systems. Probably as a consequence of a lower intracellular concentration of Fe^2+^ and S, the genes encoding superoxide dismutases (SODs) and FeS assembly were also downregulated. Please refer to Fig. 2B for details of the genes involved.

The most conspicuous alteration in gene expression was noted in the genes encoding transport systems (Fig. 8), indicating that cation homeostasis in oligotrophic environments is even more important at low temperatures. The downregulation of uptake systems and upregulation of efflux systems may lead to a decrease in the concentration of divalent cations, namely, Fe^2+^, SO_4_^2−^, Ni^2+^, and Co^2+^, as well as toxic species such as Cd^2+^ and harmful molecules. Our results showed that the cold-induced downregulation of the Fur-regulated genes involved in iron homeostasis is mediated by Fur, perhaps to prevent oxidative stress. Several transport systems and enzymes for the assimilation and interconversion of nitrogen species were upregulated, suggesting an increase in the accumulation of NH_4_^+^. The preferred inorganic nitrogen source of the cell is NH_4_^+^, which can also be obtained by nitrate/nitrite reduction and in turn incorporated into glutamate by glutamine synthase (GlnA), generating glutamine. The C. crescentus NA1000 genome has three annotated *glnA* genes, *glnA2* (CCNA_03230) being 2-fold upregulated. Moreover, the glutamate synthase *gltDB* genes were downregulated, suggesting that this increase in intracellular [NH_4_^+^] could result in the accumulation of free ammonium ions in the cell, probably to keep pH homeostasis. This, in turn, could inhibit the PTS^Ntr^ relay, limiting (p)ppGpp and delaying the progression of the cell cycle (56, 65, 66). This is also in agreement with the upregulation of the cell-cycle-regulated sRNA CcnA, which also leads to a decrease in the growth rate (52).

Potassium is the main cation present within the bacterial cell, acting together with the compatible solute group, such as trehalose, to regulate osmolarity and other processes such as enzymatic activity and antifreezing resistance (67). C. crescentus does not synthesize trehalose and therefore must rely on a more restricted set of osmoregulatory compounds. The upregulation of the *kdpFABCDE* operon at low temperature indicates that intracellular K^+^ concentration is adjusted in response to cold. In K^+^-deficient environments, the high-affinity KdpFABC complex uses ATP to pump K^+^ into the cell. Interestingly, the low-affinity K^+^ transporters KefB and KefC were not differentially expressed under our conditions.

While the levels of K^+^ respond mainly to a shift in osmolarity, previous studies have reported that in Pseudomonas putida, *kdpE* is induced during cold adaptation, and in Salmonella enterica a *kdpA* mutant strain showed reduced long-term survival at low temperature after dehydration, suggesting that the importance of potassium homeostasis in low-temperature adaptation is spread among bacteria (68, 69). The *kdpFABCDE* operon is regulated at several levels, being activated by the two-component system KdpD/KdpE in response to low K^+^ levels and other signals (70, 71). A second regulatory system described in E. coli is via the nitrogen phosphotransferase system (PTS^Ntr^), which is able to sense the cellular levels of nitrogen; under N-rich conditions, the nonphosphorylated PtsN binds to KdpD, which activates the expression of the *kdp* operon (72). The use of an ATP-driven high-affinity potassium transporter can be an adaptation to the oligotrophic condition of the environment but may cause a lower energetic efficiency, which leads to several alterations in the cell. Interestingly, the Kdp system also transports NH_4_^+^ when it is at a high concentration in the medium, leading to a futile cycle that leads to the acidification of the cytoplasm (73). The induction of these systems at low temperatures suggests that under these conditions, nutrient availability is perceived differently, perhaps as a result of the osmotic changes in the surrounding medium.

In C. crescentus, the *kdp* operon is regulated by the ECF-type sigma factor σ^T^ in response to osmotic stress (45), but the induction of *kdpA* both in response to cold as well as to low [K^+^] is not dependent on SigT (Fig. 4A and B), suggesting that there is a second promoter yet to be identified that could be regulated by KdpDE. Moreover, *kdpA*, and probably the whole *kdpFABCDE* operon, are posttranscriptionally regulated via transcript stability, which is affected by RhlE. The increase in transcript stability at low temperatures has been proposed as the main regulatory mechanism of cold-induced genes (23, 36, 74, 75), but transcript stabilization is observed for many genes under this condition. A second putative mechanism that could explain the *kdpA* transcript levels being much higher in the *rho* mutant even when the mRNA is less stable is transcription termination at a Rho-dependent terminator, which could also explain the abrupt decrease in RNA-Seq reads at the end of *kdpF*. In fact, the *kdp* operon is upregulated in bicyclomycin-treated E. coli cells, suggesting that Rho-mediated attenuation is a more general mechanism for the regulation of this operon (76).

In this study, we have characterized the cold-stress response in an oligotrophic free-living bacterium. Our results showed that there is a downshift in the central metabolism, leading to a slow growth mode, together with a decrease in the iron metabolism and the assimilatory sulfate reduction. On the other hand, the upregulation of transport systems, leading to an increase in cytoplasmic NH_4_^+^ and K^+^, suggests the need for adjusting the pH and osmotic homeostasis of the cytoplasm. The expressive number of transporters and efflux systems that were induced suggests that the response to cold stress is also increasing the resistance to other stresses, such as toxic compounds and drugs. The upregulation of genes for both transcriptional and posttranscriptional regulatory networks showed that the fine tuning of the cold stress response is complex and involves several layers of regulation.

## MATERIALS AND METHODS

### Strains and growth conditions.

The C. crescentus and E. coli strains are described in Table S2. C. crescentus NA1000 (77) was used as the wild-type strain for all experiments. C. crescentus cultures were grown at 30°C with agitation at 250 rpm in M2 minimal medium (78). E. coli strains were grown in LB complex medium. When necessary, kanamycin was then added to the cultures: 5 μg/ml for C. crescentus and 50 μg/ml for E. coli.

Growth at low [K^+^] was carried out in a sodium phosphate-buffered M2 medium (M2-K), where sodium phosphate substituted for potassium phosphate, and different final K^+^ concentrations were obtained by adding KCl back to the medium. To establish the [K^+^] concentration at which the mutant phenotype was most pronounced, the inoculum was grown in M2 medium overnight, washed twice in M2-K, and the cells were diluted to an optical density at 600 nm (OD_600_) of 0.1 in 15-ml tubes in 2 ml M2-K with different [K^+^] concentrations. The growth of NA1000 and *kdpA* strains was determined after incubation for 24 h at 30°C with agitation. Once the best [K^+^] concentration was determined, the growth was measured every 15 min in M2-K containing either 1 mM or 100 μM KCl in 2-ml cultures in 12-well plates incubated in the SpectraMax Paradigm device (Molecular Devices) with agitation at 30°C. Growth at 10°C was carried out the same way, except that the plates were incubated in an orbital shaker at 10°C and the OD_600_ value was measured in the device every 4 h.

For the RNA-Seq and RT-qPCR experiments, six 15-ml NA1000 cultures were grown in M2 in Erlenmeyer flasks at 30°C with agitation at 250 rpm until the OD_600_ value reached 0.5. Three cultures were collected and maintained at –80°C until RNA extraction, and three cultures were transferred to a shaker at 10°C and incubated with agitation at 250 rpm for 2 h before harvesting. The OD_600_ values of the cultures after this period at 10°C were very similar (0.6). The RNAs were obtained from three independent biological replicates at each temperature as described below. For RT-qPCR, the NA1000, *sigT*, and *kdpA* cultures were grown in M2 medium until the OD_600_ value equalled 0.3 to 0.4 and washed twice in M2-K; the cells were resuspended in the same volume of M2-K containing either 1 mM or 50 μM KCl. After incubation for 2 h at 30°C with agitation, the cells were collected by centrifugation for RNA extraction.

### Total RNA extraction and sequencing (RNA-Seq).

Total RNA was extracted from exponential phase (OD_600_ = 0.5) cultures grown in the same batch of M2 minimal medium at 30°C and then transferred or not to 10°C for 2 h. For RT-qPCR experiments, total RNA was extracted from 1-ml cultures from two independent biological replicates using the TRIzol Reagent (Invitrogen Life Technologies), following the manufacturer’s instructions. The RNA concentration after extraction was determined using the OD_260/280_ ratio, and the integrity was confirmed by electrophoresis. The samples were maintained at –80°C until use.

For the RNA-Seq experiments, total RNA was extracted from 2-ml cultures from three independent biological replicates using an RNeasy minikit (Qiagen), and the RNA concentration and integrity were verified using a 2100 Bioanalyzer instrument (Agilent Technologies, Waldbronn, Germany). RNA-Seq libraries were prepared using the Illumina stranded total RNA ligation with a RiboZero Plus kit (Illumina) and sequenced using the NextSeq 500/550 midoutput kit v2.0 (150 cycles) (Illumina) on the Illumina NextSeq 500 platform.

### RNA-Seq and functional enrichment analyses.

We processed the RNA-Seq raw data using the frtc pipeline (available at https://github.com/alanlorenzetti/frtc/) (43). Then, we performed differential expression analysis using DESeq2 (79) (available at https://github.com/alanlorenzetti/ccrescentus_RNASeq_analysis). A detailed description of the whole analysis is available in the supplemental material (RNASeq data analysis).

We carried out COG category enrichment analysis by checking for the overrepresentation of categories within the set of upregulated and downregulated genes. In summary, for each category, we performed the hypergeometric test implemented in the stats::phyper function of R and adjusted the computed *P* values using the Benjamini-Hochberg correction method implemented in the stats::p.adjust function of the same statistical suite.

### Reverse transcription quantitative real-time PCR.

Two micrograms of total RNA from each sample was treated with one unit of DNase I (Invitrogen) and tested for the absence of genomic DNA by PCR using primers for the *rho* gene (Table S3). Single-strand cDNA synthesis was performed using the SuperScript III first-strand synthesis kit for RT-PCR (Invitrogen). Quantitative real-time PCR experiments were performed using Power SYBR green and PCR master mix (Applied Biosystems) with the respective primer pairs (Table S3) and the CCNA_02070 gene as a reference control. The reactions were performed in duplicate for each biological replicate in a StepOnePlus real-time PCR system (Thermo Fisher Scientific). The relative change in the expression of each gene was calculated using the 2^-ΔΔCt^ relative expression quantification method (80).

### mRNA decay experiments.

Cultures from strains NA1000 (wt), *rho*::Tn5, and *rhlE*::Tn5 were grown in M2 medium at 30°C with agitation until the OD_600_ value reached 0.5. Samples (1 ml) were centrifuged for 1 min; the pellets were resuspended in 0.5 ml of TRIzol Reagent (Invitrogen Life Technologies) and frozen in a dry ice/ethanol bath (t_0_). Then, 200 μg/ml rifampin (Sigma-Aldrich) was added to each culture kept at 30°C with agitation; samples were taken at several time points and immediately treated and frozen in the same manner. Determination of the mRNA decay at 10°C was carried out in the same way, except that the cultures were incubated for 2 h at 10°C before taking the t_0_ aliquot and adding rifampin.

Total RNA was isolated and converted to cDNA as described above. The mRNA decay was assessed by RT-qPCR using equal amounts of cDNA for each point, with primer pairs for the CCNA_02070 and *kdpA* genes (Table S3).

### Construction of the Δ*kdpA* strain.

The *kdpA* gene was removed from the C. crescentus chromosome by an in-frame deletion generated by double homologous recombination. Fragments corresponding to the flanking regions of the *kdpA* gene were obtained via PCR amplification using the primer pairs kdpA IF1/kdpA IF2 and kdpA IF3/kdpA IF4 (Table S3). These fragments were then cloned into the pNPTS138 suicide vector (gift of M.R.K. Alley) using the In-Fusion HD cloning kit (TaKaRa Bio) and introduced into competent E. coli DH10B cells. The confirmed recombinant vector was then transformed into the conjugative strain E. coli S17-1 (81) and transferred to C. crescentus NA1000 through conjugation. The resulting colonies, containing the vector integrated into their chromosomes via homologous recombination, were selected for kanamycin resistance. Enrichment for the second recombination was obtained by growth in PYE medium for 48 h, followed by plating in PYE with 3% sucrose. The Kan-sensitive clones were analyzed using PCR with the primers del-Fw/del-Rv (Table S3), and a confirmed clone presenting a 0.65-kb fragment (Fig. S5) was the *kdpA*-deleted mutant selected for further characterization.

### Statistical analyses.

The graphs show the average of the number of experiments indicated in each figure, and the bars indicate the standard deviation. The statistical significance was calculated pairwise using Student’s *t* test.

### Data availability.

The raw RNA-Seq data generated in this study are publicly available at NCBI’s Sequence Read Archive (SRA) under accession number SRP310957. Detailed information about the samples and a project overview are available at NCBI under BioProject accession number PRJNA714975.
