# Supplementary material for: Cold Regulation of Genes Encoding Ion Transport Systems in the Oligotrophic Bacterium Caulobacter crescentus
Source: Microbiol Spectr. 2021 Aug 25;9(1):10.1128/spectrum.00710-21. doi: 10.1128/spectrum.00710-21 (PMC8552747; doi:10.1128/spectrum.00710-21)
Supplement: SUPPLEMENTAL FILE 2 — Supplemental material. Download SPECTRUM00710-21_Supp_2_seq11.pdf, PDF file, 0.5 MB [file spectrum00710-21_supp_2_seq11.pdf]

Supplemental material for manuscript:

Cold regulation of genes encoding ion transport systems in the oligotrophic bacterium *Caulobacter crescentus*

Hugo L. de Araújo[a], Bianca P. Martins[a], Alexandre M. Vicente[a*], Alan P.R. Lorenzetti[b], Tie Koide[b], Marilis V. Marques[a#]

SUPPLEMENTAL MATERIALS AND METHODS

RNA-Seq data analysis

We processed RNA-Seq raw data using the frtc pipeline (available at https://github.com/alanlorenzetti/frtc/) (1). Briefly, we inspected the quality of reads using Rqc (2); trimmed adapters, the first base of reads (according to manufacturer instructions), and remaining low quality ends (Q < 30) using Trimmomatic (3); aligned trimmed reads to the reference genome (NCBI Assembly ASM2200v1; RefSeq version) using HISAT2 without splicing (4); and generated read depth visualization using deepTools2 (5). We performed quality control inspection of transcriptional signal using IGV (6).

We carried out differential expression analysis using a custom set of scripts (available at https://github.com/alanlorenzetti/ccrescentus_RNASeq_analysis). We performed counting of reads per annotated feature using GenomicAlignments (7), and differential expression analysis using DESeq2 (8) with group design (NA1000 10ºC vs. NA1000 30ºC). The thresholds established to call differentially expressed genes were $\log_2$ fold change ≥ 1 or ≤ -1 and adjusted p-value < 0.01. Furthermore, we retrieved functional annotation from KEGG (9) using KEGGREST, and from UniProt (10) using UniProt.ws.

We also retrieved Clusters of Orthologous Groups (COG) (11) data for *Caulobacter crescentus* CB15 and extended the functional annotation to orthologs in *Caulobacter crescentus* NA1000 according to OrthologeDB (12).

Phenotypic tests

The cultures were grown in either PYE or M2 medium at 30°C to mid-log phase ($OD_{600\ nm}$ = 0.4-0.5) before submitted to each stress and the *kdpA* strain survival was compared to that of the wild-type NA1000 strain. The resistance to osmotic stress was evaluated by adding NaCl to 1 ml-cultures to a final concentration of 85 mM and sucrose to 150 mM. The cultures were further incubated for 2h before plating serial dilutions into PYE medium. To measure the resistance to acid stress in PYE, cells from 1 ml-cultures were pelleted by centrifugation (1 min 11,000 x *g*), the medium was discarded and the cells were resuspended in the same volume of PYE medium adjusted to pH 4.0. The cultures were then incubated for 10 min before plating serial dilutions into PYE medium. The resistance to low temperature was evaluated by plating serial dilutions of each culture and incubating the plates at 15°C for five days. All experiments were performed in duplicates.

Table S2: Strains used in this work

	Strain	Description	Reference
Caulobacter crescentus	NA1000	Wild-type strain	(13)
	SP3710 (*rho*::Tn5)	Impaired function *rho* mutant strain	(14, 15)
	rhlE::Tn5	Null *rhlE* strain	(16)
	Δ*sigT*	Deleted ECF factor sigmaT strain	(17)
	Δ*kdpA*	Deleted *kdpA* strain	This study
	Δ*fur*	Deleted *fur* strain	(18)
E. coli	DH10B	Cloning strain	(19)
	S17-1	Conjugative strain	(20)

Table S3: Primers used in this work

qRT PCR primers

Entry code	Primer sequence
**CCNA_02070**	Fw: 5' CTGACACTGTCTCGCTGGAA<br>Rv: 5' CGGAAAGCTATCCAGCGTGT
**Rho (CCNA_03876)**	Fw: 5' GTC GAG AAC GCC AAC TCC AT 3'<br>Rv: 5' CGA GGG TCT TCA GGA TCG C 3'
**KdpA (CCNA_01663)**	Fw: 5' CTGGTGGAACGTGACTCTGG 3'<br>Rv: 5' GATCAGGAGGCCGATGAAG 3'
**RhlB (CCNA_01923)**	Fw: 5' TGATGACCGCGTGCAGTTCCTC 3'<br>Rv: 5' GCTTCTTGGGCGGCGTCATCTTG 3'
**CCNA_00979**	Fw: 5' GAGGGCGATCAGGACCTTTC 3'<br>Rv: 5' GACCACATGGACCTTGGGC 3'
**CspA (CCNA_02997)**	Fw: 5' CCGTGAAGTGGTTCAACTCC 3'<br>Rv: 5' CTGACCTTCGTTCGTTCAGCGAG 3'

**DbpA (CCNA_01546)**	Fw: 5' CCTCGACCGAAGAGGATGTC 3'<br>Rv: 5' CGTCATAGATGTCTTCCGGC 3'
**NtrB (CCNA_01814)**	Fw: 5' TGAGCCCGTCAATATCCACC 3'<br>Rv: 5' CGTAGCTTTCCTTCAGCGTC 3'
**NirB (CCNA_00651)**	Fw: 5' ACTGAGGATGAAGCGGTCTG 3'<br>Rv: 5' TCCATCCACTTGTAGACCCG 3'
**RhlE (CCNA_00878)**	Fw: 5' AACTGGCGACGCAAATCG 3'<br>Rv: 5' CATCTGCGGGCCGTACTTC 3'
**NasC (CCNA_00653)**	Fw: 5' AGGTGCGAGGAACACGTAC 3'<br>Rv: 5' CCATACAAAACCCGTCATCCC 3'
**CCNA_03218**	Fw: 5' ACGGTCAAGGTCGATGAGATC 3'<br>Rv: 5' AAAGCTCGATCTCGACCAGAAC 3'
**R0094**	Fw: 5' TTGCTCGCCTATCATCCCC 3'<br>Rv: 5' CTCTGACCGGACTCGTTCC 3'
**FeoA (CCNA_00748)**	Fw: 5' CGTCGTCTGCTGGAGATGGG 3'<br>Rv: 5' GAATTTCACGCTGATCGCACCG 3'
**CCNA_02277**	Fw: 5' ATTCACGCCGACCGCAAATC 3'<br>Rv: 5' CGTCAGGTTGAAGACGCCTG 3'
**CCNA_03023**	Fw: 5' CTGGACGCGCTTTGACCTC 3'<br>Rv: 5' CCCAGCGTCAGGTAGTTGG 3'
**CCNA_00028**	Fw: 5' CGCCTATACCAGCGTCAACC 3'<br>Rv: 5' CTCTTCGACACATGGTAGGC 3'
**R0138**	Fw: 5' AGGTGCGAGGAACACGTAC 3'<br>Rv: 5' CCATACAAAACCCGTCATCCC 3'

***ΔkdpA* construction**

**Primer name**	**Primer sequence**
kdpA IF1	5' GATATCTGGATCCACGAATTCCAAAAGCAAGGTTCGACGC 3'
kdpA IF2	5' AGCGGAGGGCTTGCCATGTCATGGAATTGGTCC 3'

kdpA IF3	5’ GACATGGCAAGCCCTCCGCTGAGTGGTGATCC 3’
kdpA IF4	5’ ACGGCCGAAGCTAGCGAATTGGACATTGACCTTCAAGAGGCGG 3’
Del Fw	5’ GGATTCAAATACGCCCGGCGACGTCTTCC 3’
Del Rv	5’ GGATCCAGGTTCCGGTGTTGG 3’

SUPPLEMENTAL FIGURES

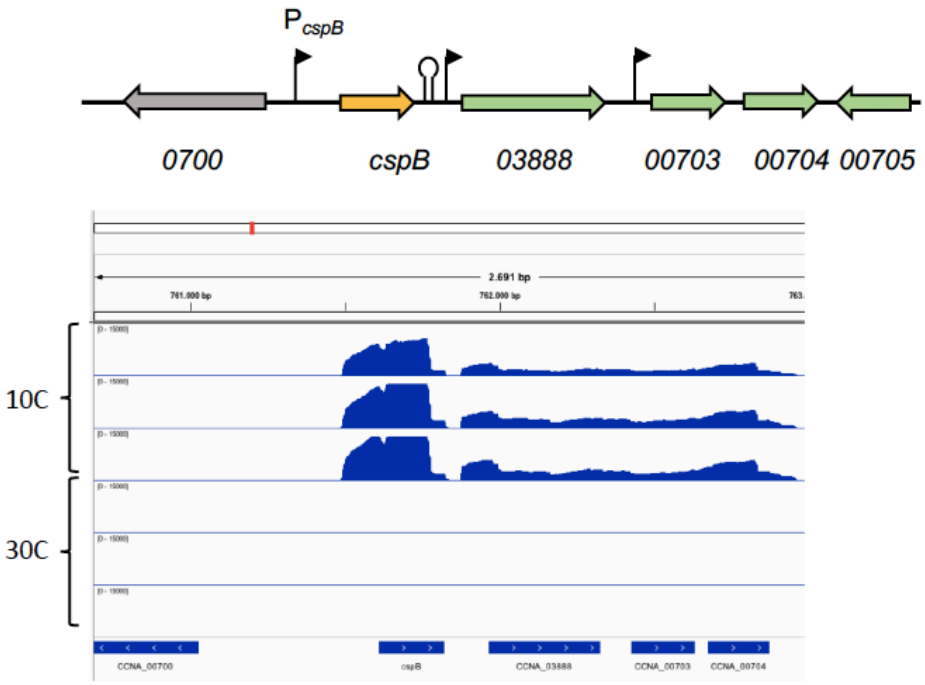

Figure S1. Analysis of the genomic region of the *cspB* gene. The map shows the *cspB* locus, indicating the position of the *cspB* promoter (bent arrow P_{cspB}) and intrinsic terminator (stem loop). Downstream of *cspB*, the CCNA_03888 gene and the CCNA_00703/CCNA_00704 operon are transcribed independently, from putative promoters (bent arrows). Below is shown the read coverage of the region downstream to the *cspB* gene. Each line corresponds to the read counts of the RNA-Seq of three biological replicates at 30°C and three at 10°C as indicated. The graphs were generated using the Integrative Genomics Viewer (6) to observe RNA-Seq BEDGRAPH files.

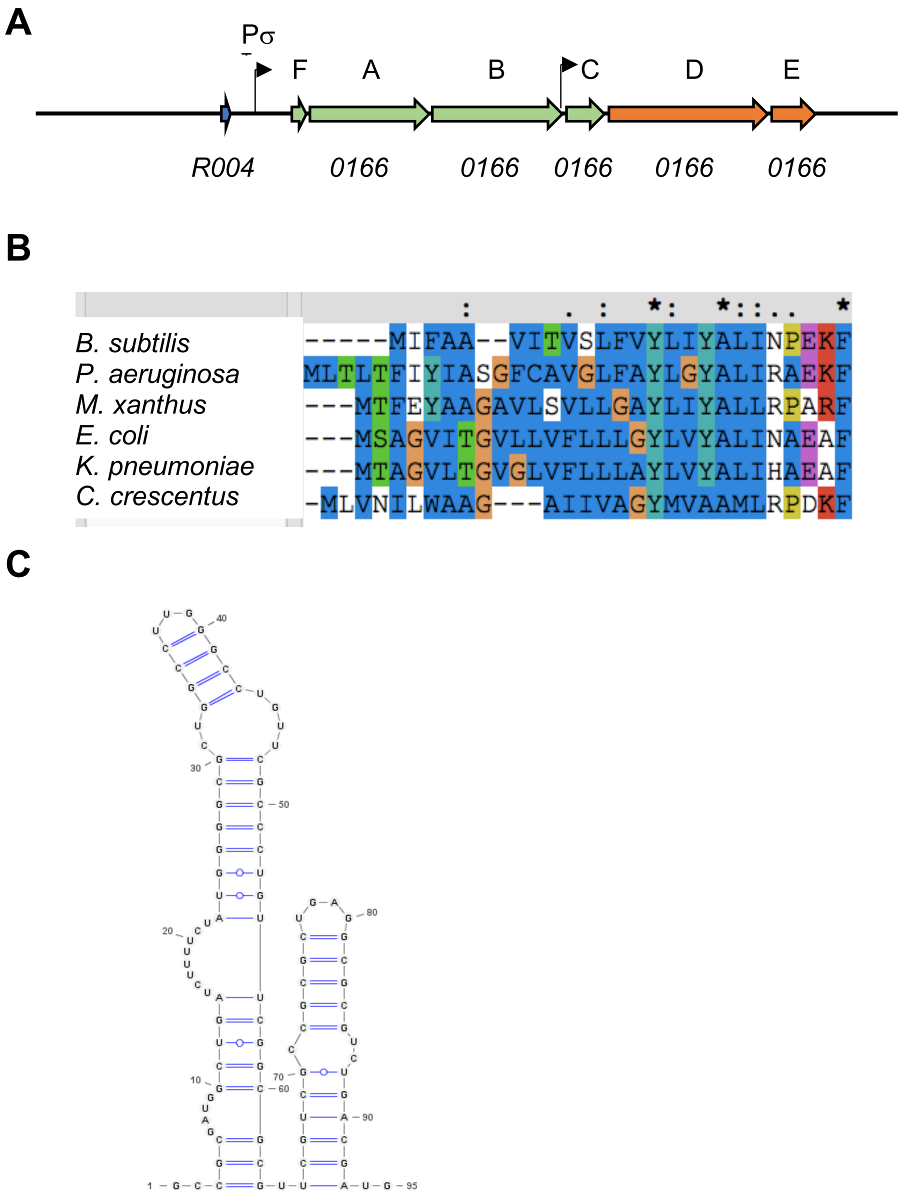

Figure S2. Analysis of the 5' region of the *kdp* operon. A. Map of the *kdp* operon, indicating the position of *kdpF* upstream of *kdpA*. A putative sigmaT promoter (P_{sigT}) is located upstream to *kdpF*, and a putative second promoter is probably located upstream of *kdpC* (bent arrows). B. Amino acid sequence alignment of KdpF from selected bacteria and the deduced amino acid sequence of the ORF upstream of *kdpA* (*C. crescentus*). Alignment was carried out with Clustal X (21), using the sequences: YP_588443.1 (*Escherichia coli* MG1655); AAK81842.1 (*Myxococcus xanthus*); QHH22443.1 (*Bacillus subtilis*); QDC84763.1 (*Klebsiella pneumoniae*); WP_069075609.1 (*Pseudomonas*). C. Prediction of the secondary structure of the 5' untranslated region of *kdpF*. The sequence corresponds to the region 10 nt downstream of the PsigT promoter until the start codon of KdpF. Structure was predicted using the RNAfold suite (http://rna.tbi.univie.ac.at/cgi-bin/RNAWebSuite/RNAfold.cgi) (22) and drawn with the VARNA software (http://varna.lri.fr/) (23).

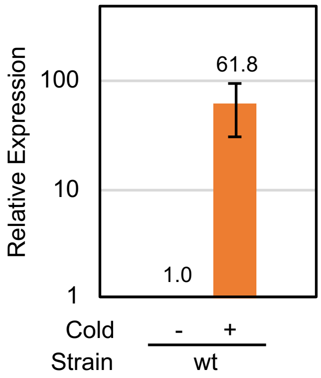

Figure S3. **Relative *kdpA* mRNA levels in *C. crescentus***. Expression was determined by RT-qPCR from total RNA isolated from cultures growing in in PYE medium at 30°C (Cold -) and after 2h at 10°C (Cold +). Expression was determined relative to the wt strain at 30°C.

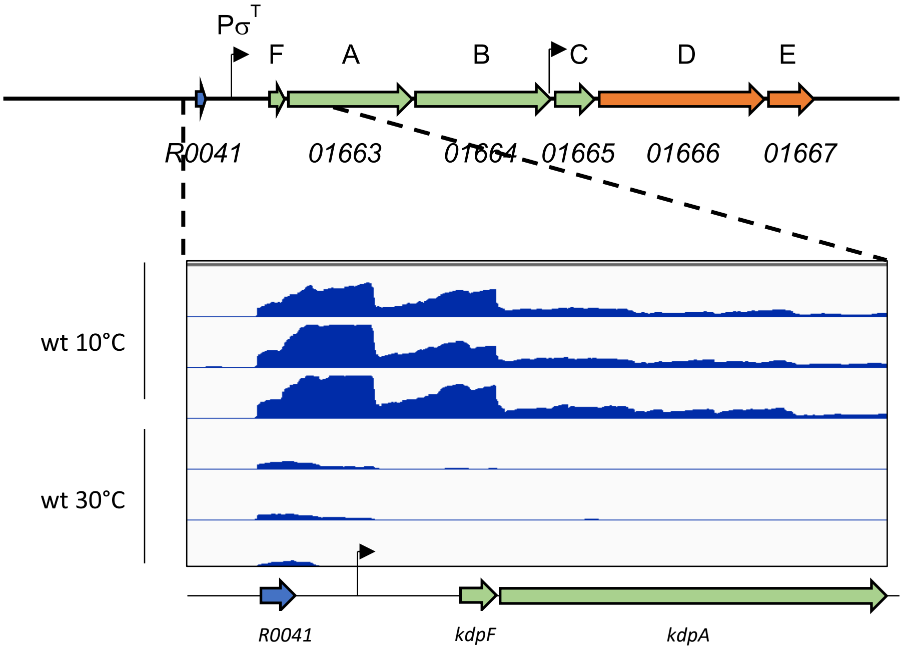

Figure S4. Read coverage of the region upstream to the *kdp* operon. The region comprised between the R0041 gene and the beginning of the *kdpA* gene is shown. Each line corresponds to the read counts of the RNA-Seq of three biological replicates at 30°C and three at 10°C as indicated. The graphs were generated using the Integrative Genomics Viewer (6) to observe RNA-Seq BEDGRAPH files. The position of the ORFs is shown below, and the bent arrow indicates the P_{sigT} promoter.

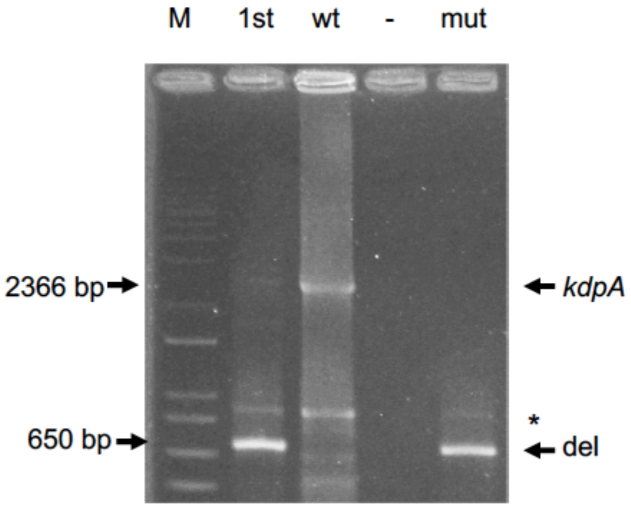

Figure S5. Confirmation of the *kdpA* deletion. Total genomic DNA from strains NA1000 (wt), first recombinant (1st) and deleted mutant (mut) were used as template in a PCR reaction using primers flanking the *kdpA* gene. The bands corresponding to the complete gene (*kdpA*) and the deleted gene (del) are indicated. M stands for molecular weight marker (1 kb Plus, Thermo Fisher Scientific). Negative sign (-), negative control containing no template DNA. The asterisk indicates a non-specific band.

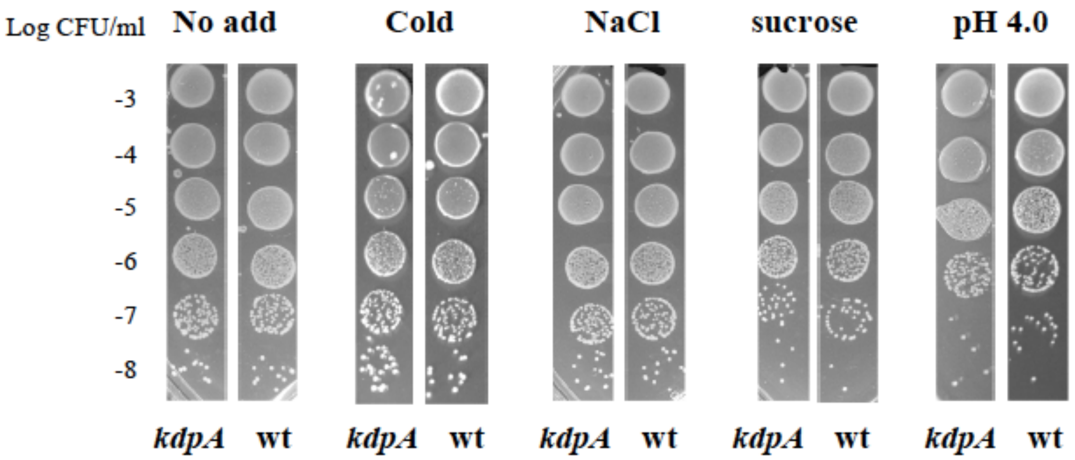

Figure S6. Phenotypic characterization of the *kdpA* mutant. Phenotypic analysis of the *kdpA* mutant strain in PYE medium. Mid-log phase cultures of wild-type (NA1000) and *kdpA* strains were used to ascertain the resistance to several stresses. The resistance to low temperature (Cold) was evaluated by plating serial dilutions of each culture into PYE medium and incubating the plates at 15°C for five days. For osmotic stress, the cultures were incubated with 85 mM NaCl (NaCl) or 150 mM sucrose (Sucrose) for 2h before plating into PYE medium. For acidic stress, the cells grown in PYE medium were centrifuged and resuspended in the same volume of PYE medium adjusted to pH 4.0 for

10 min before plating (pH 4.0). Serial dilutions are indicated as log_{10}(CFU/ml). All the experiments were done in biological duplicates and a representative result is shown.

REFERENCES:

1. Ten-Caten F, Vêncio RZN, Lorenzetti APR, Zaramela LS, Santana AC, Koide T. 2018. Internal RNAs overlapping coding sequences can drive the production of alternative proteins in archaea. RNA Biol. 15:1119-1132.
2. Souza, W, Carvalho BS, Lopes-Cendes I. 2018. Rqc: A Bioconductor Package for Quality Control of High-Throughput Sequencing Data. Journal of Statistical Software 87: 1–14
3. Bolger AM, Lohse M, Usadel B. 2014. Trimmomatic: A Flexible Trimmer for Illumina Sequence Data. Bioinformatics 30: 2114–20.
4. Kim D, Paggi JM, Park C, Bennett C, Salzberg SL. 2019. Graph-Based Genome Alignment and Genotyping with HISAT2 and HISAT-Genotype. Nature Biotechnol 37: 907–15.
5. Ramírez F, Ryan DP, Grüning B, Bhardwaj V, Kilpert F, Richter AS, Heyne S, Dündar F, Manke T. 2016. DeepTools2: A next Generation Web Server for Deep-Sequencing Data Analysis. Nucleic Acids Res 44: W160-165.
6. Thorvaldsdóttir H, Robinson JT, Mesirov JP. 2013. Integrative Genomics Viewer (IGV): High-Performance Genomics Data Visualization and Exploration. Briefings in Bioinformatics 14: 178–92.
7. Lawrence M, Huber W, Pagès H, Aboyoun P, Carlson M, Gentleman R, Morgan MT, Carey VJ. 2013. Software for Computing and Annotating Genomic Ranges. PLoS Comput Biol 9.
8. Love MI, Huber W, Anders S. 2014. Moderated Estimation of Fold Change and Dispersion for RNA-Seq Data with DESeq2. Genome Biol 15.
9. Kanehisa M, Furumichi M, Tanabe M, Sato Y, Morishima K. 2017. KEGG: New Perspectives on Genomes, Pathways, Diseases and Drugs. Nucleic Acids Res 45: D353–61.
10. The UniProt Consortium. 2021. UniProt: The Universal Protein Knowledgebase in 2021. Nucleic Acids Res 49: D480–89.
11. Galperin, MY, Makarova KS, Wolf YI, Koonin EV. 2015. Expanded Microbial Genome Coverage and Improved Protein Family Annotation in the COG Database. Nucleic Acids Res 43: D261–69.
12. Whiteside MD., Winsor GL, Laird MR, Brinkman FSL. 2013. OrtholugeDB: A Bacterial and Archaeal Orthology Resource for Improved Comparative Genomic Analysis. Nucleic Acids Res 41: D366-376.
13. Evinger M, Agabian N. 1977. Envelope-associated nucleoid from *Caulobacter crescentus* stalked and swarmer cells. J Bacteriol, 132: 294-301.
14. Italiani VC, Marques MV. 2005. The transcription termination factor Rho is essential and autoregulated in *Caulobacter crescentus*. J Bacteriol 187:4290-4294.
15. Italiani, VCS, Zuleta LFG, Marques MV. 2002. The transcription termination factor Rho is essential for oxidative stress survival in *Caulobacter crescentus*. Mol Microbiol 44:181–194.

16. Mazzon RR, Lang EA, Braz VS, Marques MV. 2008. Characterization of *Caulobacter crescentus* response to low temperature and identification of genes involved in freezing resistance. FEMS Microbiol Lett 288: 178-185.

17. Alvarez-Martinez CE, Lourenço RF, Baldini RL, Laub MT, Gomes SL. 2007. The ECF sigma factor sigma(T) is involved in osmotic and oxidative stress responses in *Caulobacter crescentus*. Mol Microbiol. 66:1240-55.

18. da Silva Neto JF, Braz VS, Italiani VCS, Marques M V. 2009. Fur controls iron homeostasis and oxidative stress defense in the oligotrophic alpha-proteobacterium *Caulobacter crescentus*. Nucleic Acids Res 37:4812–4825.

19. Grant SG, Jessee J, Bloom FR, Hanahan D. 1990. Differential plasmid rescue from transgenic mouse DNAs into *Escherichia coli* methylation-restriction mutants. Proc Natl Acad Sci USA 87:4645–4649.

20. Simon R, Priefer U, Pühler A. 1983. A broad host range mobilization system for in vivo genetic engineering: transposon mutagenesis in gram negative bacteria. Biotechnology, 1: 784-791.

21. Larkin MA, Blackshields G, Brown NP, Chenna R, McGettigan PA, McWilliam H, Valentin F, Wallace IM, Wilm A, Lopez R, Thompson JD, Gibson TJ, Higgins DG. 2007. Clustal W and Clustal X version 2.0. Bioinformatics, 23:2947-2948.

22. Lorenz R, Bernhart SH, Siederdissen CHZ, Tafer H, Flamm C, Stadler PF, Hofacker IL. 2011. ViennaRNA Package 2.0. Algorithms for Molecular Biology: AMB 6: 26.

23. Darty K, Denise A, Ponty Y. 2009. VARNA: Interactive drawing and editing of the RNA secondary structure. Bioinformatics 25:1974-1975.
